# Two years of school-based intervention program could improve the physical fitness among Ecuadorian adolescents at health risk: subgroups analysis from a cluster-randomized trial

**DOI:** 10.1186/s12887-016-0588-8

**Published:** 2016-04-22

**Authors:** Susana Andrade, Carl Lachat, Greet Cardon, Angélica Ochoa-Avilés, Roosmarijn Verstraeten, John Van Camp, Johana Ortiz, Patricia Ramirez, Silvana Donoso, Patrick Kolsteren

**Affiliations:** Food Nutrition and Health Program, Universidad de Cuenca, Avenida 12 de Abril y Loja, 010202 Cuenca, Ecuador; Department of Food Safety and Food Quality, Ghent University, Coupure links 653, 9000 Ghent, Belgium; Institute of Tropical Medicine, Nationalestraat 155, 2000 Antwerp, Belgium; Department of Movement and Sports Sciences, Ghent University, Watersportlaan 2, 9000 Ghent, Belgium

**Keywords:** Fitness, Physical activity, Adolescents, Randomized control trial, Subgroup analysis, Body mass index

## Abstract

**Background:**

Adolescents with overweight and poor physical fitness have an increased likelihood of developing cardiovascular diseases during adulthood. In Ecuador, a health promotion program improved the muscular strength and speed-agility, and reduced the decline of the moderate-to-vigorous physical activity of adolescents after 28 months. We performed a sub-group analysis to assess the differential effect of this intervention in overweight and low-fit adolescents.

**Methods:**

We performed a cluster-randomized pair matched trial in schools located in Cuenca–Ecuador. In total 20 schools (clusters) were pair matched, and 1440 adolescents of grade 8 and 9 (mean age of 12.3 and 13.3 years respectively) participated in the trial. For the purposes of the subgroup analysis, the adolescents were classified into groups according to their weight status (body mass index) and aerobic capacity (scores in the 20 m shuttle run and FITNESSGRAM standards) at baseline. Primary outcomes included physical fitness (vertical jump, speed shuttle run) and physical activity (proportion of students achieving over 60 min of moderate–to-vigorous physical activity/day). For these primary outcomes, we stratified analysis by weight (underweight, normal BMI and overweight/obese) and fitness (fit and low fitness) groups. Mixed linear regression models were used to assess the intervention effect.

**Results:**

The prevalence of overweight/obesity, underweight and poor physical fitness was 20.3 %, 5.8 % and 84.8 % respectively. A higher intervention effect was observed for speed shuttle run in overweight (β = −1.85 s, *P* = 0.04) adolescents compared to underweight (β = −1.66 s, *P* = 0.5) or normal weight (β = −0.35 s, *P* = 0.6) peers. The intervention effect on vertical jump was higher in adolescents with poor physical fitness (β = 3.71 cm, *P* = 0.005) compared to their fit peers (β = 1.28 cm, *P* = 0.4). The proportion of students achieving over 60 min of moderate-to-vigorous physical activity/day was not significantly different according to weight or fitness status.

**Conclusion:**

Comprehensive school-based interventions that aim to improve diet and physical activity could improve speed and strength aspects of physical fitness in low-fit and overweight/obese adolescents.

**Trial registration:**

Clinicaltrials.gov identifier NCT01004367. Registered October 28, 2009.

**Electronic supplementary material:**

The online version of this article (doi:10.1186/s12887-016-0588-8) contains supplementary material, which is available to authorized users.

## Background

Overweight and lack of physical fitness in adolescence are independent risk factors for the development of non-communicable diseases (NCDs) throughout the life course [1–4]. Overweight adolescents are on average 1.5 times more likely to develop type II diabetes, hypertension and an abnormal lipid profile during adulthood. Only recently, adolescents with low fitness levels are considered as a public health issue as their low fitness levels are significantly related with unhealthy cardiovascular performance, muscle mass losses, adipose tissue increase, decreased insulin response and sensitivity, and low bone mineral density in adulthood [3].

NCD prevention strategies such as school-based interventions, are particularly important since these are feasible and relatively inexpensive approaches that reach out to large populations with a wide range of BMIs or fitness abilities. School-based interventions involving both the individual and environmental components have shown small to moderate effects for the prevention of overweight and low-fitness in adolescents [5–8]. However, to our knowledge, little is known about the effect of these school-based interventions in groups of adolescents with a high health risk, like overweight/obese and low-fit adolescents. Current research on the topic is focused on the 6 to 12 year age group from high-income countries [9–15]. In low-and middle-income countries (LMICs), the evidence on the effectiveness of school based interventions for the prevention of overweight and low-fitness is limited and specifically scarce regarding to its effect modification on high-risk groups such as overweight/obese and low-fit adolescents [3, 9–14].

In Cuenca-Ecuador a school-based intervention program “ACTIVITAL”, with a sample of adolescents (*n* = 1440), was carried out. The intervention was developed by using the Intervention mapping protocol [16] together with a participatory approach (Comprehensive Participatory Planning and Evaluation approach [17]. In summary, the needs assessments include a qualitative [18, 19] and quantitative [20, 21] research which identified the influencing factor (individual and environmental) for diet and physical activity behavior [18, 19]. In the sample targeted by quantitative research, 3 out of 5 adolescents had low fitness scores [20] and the prevalence of overweight and obesity was 18 % and 2.1 %, respectively [21]. This information was used to define the intervention objectives. The objectives were translated into intervention strategies using theories reported to be effective in other studies. The developed strategies were then adapted to the local context by using the local evidence and the participatory approach (participatory workshops with school staff and adolescents). This overall process resulted in a multicomponent (individual/environmental) intervention program aimed to (i) decrease sugar intake, (ii) increase daily fruit and vegetable intake, (iii) decrease unhealthy snack intake, (iv) increase healthy breakfast intake, (v) decrease daily screen time, and (vi) increase physical activity of adolescents [18, 19, 22]. In line with these objectives, diet, physical fitness, physical activity and screen-time were defined as primary outcomes, while anthropometric measurements (body mass indices, waist circumference) and blood pressure were secondary ones. After 28 months, the intervention showed an effect on three primary outcomes, diet [23], physical fitness and physical activity [24] and on two secondary outcomes: blood pressure and waist circumference [23].

The present manuscript assessed if the adolescents in high-risk groups, specifically those overweight/obese and low-fit, responded differently to the intervention compared to their peers in lower risk groups in terms of physical fitness (speed shuttle run and vertical jump) and physical activity (the proportion of adolescents who met the recommended 60 min of moderate to vigorous physical activity per day). The subgroup analysis of dietary outcomes was presented elsewhere [23].

## Methods

The ACTIVITAL study was a pair-matched cluster randomized control trial conducted from October 2009 till June 2012 in Cuenca, an urban area in the south of Ecuador located at ±2400 m of altitude. Schools were used as clusters to avoid contamination between intervention and control arms.

### Participants, sampling, allocation and recruitment

Inclusion criteria for schools were: (i) having >90 students in 8th and 9th grade and (ii) located in the urban area of Cuenca, Ecuador. The schools were matched according to: (i) total number of the students (ii) monthly school fee (as approximation of socio-economic status of the school), (iii) school gender (male/female only or co-ed schools) and (iv) time schedule (morning: 7:00 to 13:00 or afternoon: 12:00 to 18:00). After the matching the schools without pair were excluded. A total of 28 (14 pairs) out of 108 schools fitted the inclusion criteria.

The sample size needed to detect a 10 % reduction of energy intake from fat (from 40 % to 30 % energy intake from fat, assessed using 2 × 24 h recalls [23]) in the intervention group compared to the control group was 10 pairs and 1430 adolescents. The latter was calculated based on Hayes & Bennett [25], using a statistical power of 80 %, a type I error of 5 %, a K_m_ of 0.15 and a 10 % anticipate drop-out. Stata (version 12, Stata Corporation, Texas, USA) was used to select the pairs at random and randomly allocate the intervention or control within each pair. Two 8th grades and two 9th grades were randomly selected in each school. All adolescents in the classes were invited to participate but were excluded when they were pregnant, had a muscle or bone injury or had a concomitant disease. Supervisors and interviewers were trained to carry out the measurements. Interviewers were blinded to the allocation group of the intervention and adolescents were not informed about the existence of counterfactual schools.

Coordinators of ACTIVITAL recruited adolescents, parents and schools’ principals through separate meetings. The objectives, duration and the timetable of activities of intervention were explained during the meetings. Adolescents (acceptance rate = 85 %) and their caretakers (acceptance rate = 95 %) signed a written assent and consent respectively. The principal in each school (participation rate = 100 %) formally accepted the participation of the school in the study. This study was approved by the ethics committees from Ecuador (“Comité de Biomedicina de la Universidad Central del Ecuador”, code N°: CBM/cobi-001 − 2008/462) and Belgium (“Ghent University Hospital” code N°: FWA00002482). The trial was registered under the clinicaltrials.gov as NTC01004367.

### Intervention

The intervention’s objectives and strategies were developed by a systematic process that include Intervention Mapping protocol and Comprehensive and Participatory Planning and Evaluation approach [19]. In general terms, the intervention objective was to improve the dietary and physical activity behavior and to discourage the time devoted to screen-time among adolescents. For these purposes both individual and environmental strategies were developed and implemented in two periods: October 2010 until February 2011 and from October 2011 until January 2012 (Table 1).

**Table 1:** Physical activity intervention components of the ACTIVITAL study implemented among 12–15 year old adolescents in 10 schools of Cuenca – Ecuador during 2010–2012*

What	Who/where/when	Why	How	What received (WR)/How reacted (HR)
1. Individual-based strategies				
Book 1 (Curriculum) One out of five chapters addressed physical activity and screen-tine behavior. This chapter was developed to be delivered in 90 min (1^st^ year).	School teachers and trained staff/classroom/September 2010-February 2011 Each chapter was performed every two weeks.	- To create awareness regarding the importance of an adequate physical activity throughout adolescence (Book 1 and 2) - To increase knowledge and enhance decision-making skills (Book 1 and 2) - To encourage the adolescents to be physically active for at least 60 min per day and to spend maximum 2 h per day on screen-tine activities (Book 1)	Thought textbooks and pedagogic materials for teachers and students. The material contained educational objectives, clear instructions for implementation the physical and educational activities during the classes without additional training.	WR: 100 % of classes addressing physical activity component were delivered HR: The students had a 95 % of average attendance of classes on physical activity Around 75 % of adolescents showed an active participation in the classes. Around 54 % of the scheduled classes addressing physical activity component were delivered by the school teacher
Book 2 (Curriculum) The book contained 8 chapters in total and one corresponded to the physical activity. Chapter 7: Physical Activity (how to remove barriers in order to be more physically active). This chapter was planned to be delivered in 90 min (2^th^ year).	School teachers and trained staff/classroom/September 2011-January 2012. Each chapter was performed every two weeks.		A second set of textbooks and pedagogic materials were developed for teachers and students. The material contained educational objectives and clear instructions for implementing the physical and educational activities.	
2. Environment-based strategies Parental workshops In total six workshops were performed. Informative leaflets supporting the content of the workshop were distributed to each participant during the workshops. Two workshops focused on decreasing sedentary time and increasing physical activity (1^st^ year) and dealing with barriers for physical activity (2^th^ year).	ACTIVITAL staff/school meeting room/1 workshop from October 2010 till February 2011 and 1 workshop from October 2011 till January 2012	- To support healthy behavior of adolescents at home - To increase the awareness of parents regarding the importance of regular physical activity for adolescents, how to be active during the day and how to deal with barriers to be physically active.	Workshops of 1 h were delivered by the ACTIVITAL staff. Parents attendance was mandatory through a letter signed by each school principal Each leaflet included theoretical information, advises and benefits on the particular topic of the workshops	WR: Two workshops (100 %) related to physical activity component were delivered as planned. HR: Around 10 % of the parents attended both workshops. Around 97 % of the parents showed an interest in the contents of the workshops
Social event -Pep talks by successful and well-known young male (*n* = 3) and female (*n* = 2) athletes, which were international young champions in BMX, swimming, racquetball and weightlifting (1^st^ year)	Young athletes/auditorium/Once during the intervention	- To encourage physical activity through the positive influence of social models	A 1-h interactive session with young athletes was given. Athletes shared their personal sport experiences and gave advice on active lifestyles and physical activity.	WR: One pep talk was delivered in each school (100 %) HR: Around 78 % of adolescents showed an interest in the pep talks.
Walking trail and posters - 3 posters suspended on the school walls adjacent to the trail, with phrases like: “*Do you like to talk*? *Walk and Talk*” (1^st^ year). - Using line markings, a walking trail was drawn on the school’s playground. The length of the trail was the perimeter of playground (2^th^ year).	Physical education teacher/classroom/September 2011 – January 2012	- To increase availability and accessibility to opportunities for physical activity inside the schools - To motivate the students to walk more during the recess time	The physical education teacher explained the students about the importance of being physically active and how the students could use the walking trail to be more active during recess.	WR: The walking trail was implemented in the ten schools (100 %) HR: Around 25 % of the adolescents used the walking trail according to the results of the two schools where the walking trail was evaluated.
Posters for classroom and food tuck shop Fiver different posters with key messages on physical activity and pictures of the young athletes (1^st^ year).	ACTIVITAL staff/classroom and food tuck shop/Monthly from October 2010 to February 2011	- To encourage students to be active and eat healthy	Posters included key messages to be active were suspended on the classroom walls and in front of the food tuck shops.	WR/HR: The five posters (100 %) were suspended in the classroom and food tuck shop

*The “ACTIVITAL” trial aimed at improving diet and physical activity. This table summarizes the physical activity component of the trial, which was focused on improving both physical activity and scree-time behaviors

The individual strategy included the delivery of educational package organized at classroom level to promote healthy diet and an active lifestyle. This strategy was implemented through classes for all students in the selected grades and was delivered by volunteering teachers of life sciences of the schools and research staff. The following key messages related to physical activity behavior were tackled in two out of 13 chapters of the educational package: i) be active for at least 60 min per day, ii) spend maximum 2 h per day on sedentary behavior and iii) ways to overcome the barriers for physical activity (Table 1). The other 11 chapters of the educational package focused on the promotion of a healthy diet.

The environmental strategy included three main activities: (i) Workshops for parents that were parallel to the classes with adolescents and covered similar topics (e.g. be active for at least 60 min per day, spend maximum 2 h per day on sedentary behavior and ways to overcome the barriers for physical activity). The parental workshop lasted one hour and consisted of a slide show presentation followed by a session of questions of parents. (ii) Organization of social events such an interactive pep talks with famous young sportsmen. During a one-hour session, an athlete shared her/his personal sport experiences and gave advice on healthy diet, active lifestyle and physical activity. One session per school was organized. (iii) Environmental modification that consistent of providing a walking trail in each school. Walking trails were drawn on the playground and three posters were suspended on the walls along the walking trails to encourage the adolescents to walk more during recess. Additionally, full color posters of young sportsmen, the ACTVITAL logo and key message regarding physical activity were suspended on the classroom walls and in the front of the food shops. In addition, regular meetings with schoolteachers, school management and students were held to assess progress and coordinate the intervention activities (Table 1).

Both the intervention and control schools received the standard school curriculum as determined by the Ecuadorian government, which allocates 80 min of physical education classes per week (2 school sessions). The mandatory physical education curriculum was mainly geared at increasing sports skills and was implemented in all schools by the schoolteachers.

### Measurements

The baseline and the follow-up measurements were performed October 2009-February 2010 and February 2012-June 2012 respectively. A group of interviewers (nutritionist, medical doctors and others professionals related to health, size group range: 7–14 persons) were trained for the purposes of the research (five days of training and using a manual training) and collected the data in the schools. The principals of the schools agreed to allocate a number of class hours over a one week to apply the measurements.

### Primary and secondary outcomes

According to the interventions’ objectives the primary outcomes of the trial were diet, physical fitness, sedentary behavior and physical activity, while blood pressure and anthropometric measurements were the secondary outcomes. The diet (energy intake and food group consumptions) was assessed by 24 h recalls [23]. Physical fitness was measured by EUROFIT [26] battery and included 20 m shuttle run, speed shuttle run, plate tapping, sit-and-reach, sit-ups, vertical jump, bent hang, handgrip and flamingo balance tests. As a proxy of sedentary behavior, screen time was used. The latter was estimated using a validated questionnaire [27] that assessed the time spend on television, video games and computer during a weekday (after school hours) and weekend day. Physical activity was measured using accelerometers (type GT-256 and GT1M, Actigraph Manufacturing Technology Incorporated, Fort Walton Beach FL, USA). A randomly selected subsample (acceptance rate 100 %) of adolescents (*n* = 251 at baseline, *n* = 134 after the intervention i.e. 47 % of missing data) wore an accelerometer during five weekdays. To reduce the data from accelerometer to minutes of physical activity the cut-points used were ≤100 counts/min, 100–759 counts/min and ≥760 counts/min for sedentary, light and moderate to vigorous physical activity respectively. The proportion of adolescents who met the recommended 60 min of moderate to vigorous physical activity per day [28] was calculated. The anthropometric measurements (secondary outcomes) included BMI and waist circumference, and were used to estimate changes in the anthropometric status.

As mentioned before, the present sub-group analysis considered two primary outcomes that showed a significant improvement among adolescents: physical fitness in terms of speed shuttle run and vertical jump, and physical activity in terms of the proportion of adolescents who met the recommended 60 min of moderate to vigorous physical activity per day. These outcomes showed a power >80 % based on a *post*-*hoc* analysis [25].

### Socio-economic status

The socio-economic status of the adolescent’s household was defined according to the Integrated Social Indicator System for Ecuador [29]. The system classifies a household as “poor” when it reports one or more deprivations related to housing facilities, basic urban services, money, education and physical space, otherwise the household is classified as “better-off”.

### Monitoring of delivery and response of the intervention

Researchers recorded attendance and participation rates during classes and the receptiveness of the adolescents to the classes. Teachers in charge of a class filled out a questionnaire at the end of each class to assess their appreciation of the materials and the messages conveyed. We assessed if adolescents noticed, liked and used the walking trail using a questionnaire in a convenience sample of 2 schools. At the end of the workshop with parents, a questionnaire was administered to parents to measure satisfaction and to get general feedback of the workshops. Table 1 summaries the delivery and response of the intervention. A full process evaluation is reported elsewhere [23].

A detailed description of intervention design [19], methods of collection data [24], and the intervention effect on primary outcomes dietary intake (including sub-group analysis) [23], physical fitness, physical activity [24], and screen-time (*under second revision*) can be found in a separate documents.

### Grouping

For the purpose of this paper, we classified adolescents into groups according to their BMI and aerobic capacity scores in the 20 m shuttle run at baseline. The BMI groups were normal weight, underweight and overweigh/obese (called “overweight”) and were defined according to IOTF criteria [30].

The fitness groups “fit” and “low fitness”, were generated based on the results from the 20 m shuttle run test at baseline using the FITNESSGRAM standard. The latter classifies adolescents into those who achieved the health zone (“fit group”) or not (“low fitness group”) [31]. FITNESSGRAM contains the minimum levels of aerobic capacity (in ml/kg/min units of VO2max) that provides a protection against health risks associated with inadequate fitness. For girls, standard values range from 40.2 ml/kg/min to 38.8 ml/kg/min across the developmental transition from 11 to 17 years old. For boys, values rise from around 40.2 ml/kg/min to 44.2 ml/kg/min. To obtain the VO2max from the result of the 20 m shuttle run tests the following validated equation was used VO2max = 41.77 + 0.49 (laps)-0.0029 (laps) 2-0.62 BMI + 0.35 (gender* age); where gender = 0 for girls, 1 for boys [32].

### Statistical analysis

All analyses were performed on an intention-to-treat basis. The baseline characteristics by group were presented as means with standard deviation (SD) or percentage (%). In the BMI and fitness groups we tested the differences in characteristics at baseline between categories by χ^2^ test and two-sample *t*-test, accounting for cluster design by using the STATA (command svy).

The intervention effect was analyzed using a mixed model with the pair-matching as the random factor. In such models, the Beta coefficient (β) of the intervention variable indicates the difference in means for continuous dependent variables and the difference in absolute risks for dichotomous ones [33]. We assessed whether the intervention effect varied according to BMI or fitness status by including the interaction terms BMI categorical x intervention allocation or fitness categorical x intervention allocation in the model.

All models were adjusted for gender, socio economics status and the corresponding interaction terms with intervention allocation. The model for BMI was also adjusted for fitness categorical and fitness categorical x intervention allocate, while the model for fitness was also adjusted for BMI and BMI x intervention allocation. The covariates included in the models were used as they were considered confounders. The interaction terms between covariates and intervention allocation were used to check for independent of the associations between covariates [34]. We stratified the analysis and compared the intervention effect within BMI or fitness status when the corresponding interaction term was significant based on a threshold of *P*-value of interaction (*P*_*i*_) <0.1 [34].

As a sensitivity analysis, we repeated all tests without adjusting for variables at the individual level. In addition, to estimate the effect of missing data on outcomes that were significant different among BMI or fitness status (*P*_*i*_ <0.1) we repeated the analyses after imputing missing data. Multiple imputations were done under the missing at random assumption and using the chained equation models with 50 runs of imputations. The predictors for the regression model for imputations were gender, BMI z-score, age and socio economic status at baseline since they could influence the outcome.

All statistical tests were two-sided with a statistical significance level at 5 %. Stata software (version 12.0 IC, Stata Corporation, Texas, USA) was used to perform all analyses.

## Results

### Baseline differences

**Fig. 1:**
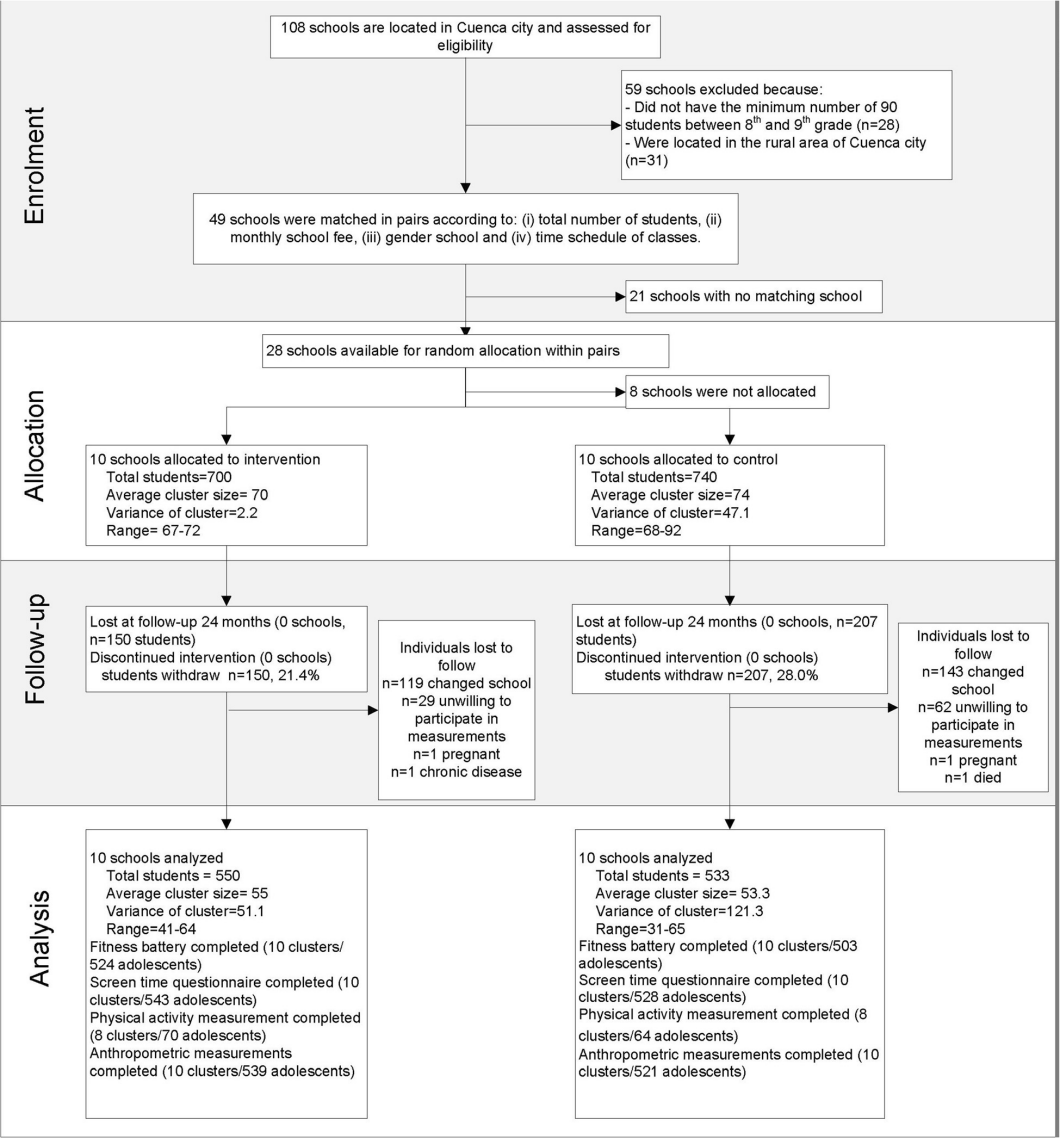
Enrolment, allocation, follow-up and analysis of Ecuadorian adolescents in a school-based health promotion intervention. ^a^The flow chart reflects the whole study population without a distinction based on their weight status and fitness [24]

The flowchart of the study is presented in Fig. 1. The baseline prevalence of overweight was 20.3 % (including 3.4 % of obese) and the underweight was 5.8 %. The largest share of the sample (84.8 %) of the adolescents were classified into the low-fit group. Only some baseline characteristics were comparable between the BMI and fitness categories. Between BMI groups, the comparable baseline characteristic were female proportion, and the proportion of adolescents who meet the PA recommendation (Table 2 and Additional file 1: Table S1). Whilst for fitness groups, only age, proportion of poor and the proportion of adolescents who meet the PA recommendation were comparable (Table 3 and Additional file 1: Table S1).

**Table 2:** Baseline characteristics by BMI status (normal weight, underweight and overweight)^a^

	*P* ^b^	All	Normal weight			Underweight			Overweight		
		*n*	Control Mean (SD)	Intervention Mean (SD)	*n*	Control Mean (SD)	Intervention Mean (SD)	*n*	Control Mean (SD)	Intervention Mean (SD)	*n*
Age	0.04	1292	12.91 (0.82)	12.80 (0.75)	1014	13.05 (0.84)	12.89 (0.84)	79	12.77 (0.78)	12.75 (0.79)	278
Body mass index (kg/m^2^)	<0.001	1292	18.79 (1.67)	18.79 (1.65)	1014	15.51 (0.83)	15.20 (0.73)	79	24.24 (2.04)	24.90 (2.81)	278
Body mass index Z-score	<0.001	1292	0.06 (0.64)	0.07 (0.68)	1014	−1.66 (0.40)	−1.80 (0.43)	79	1.73 (0.43)	1.84 (0.52)	278
Low socio economic status (%)	0.03	1240	34.56	32.78	971	29.41	41.46	75	26.36	25.00	269
Female proportion (%)	0.78	1292	58.30	66.73	1014	61.76	60.00	66	58.52	68.53	278
Fitness (EUROFIT)											
*Speed*-*agility*											
Speed shuttle run (s)	<0.001	1257	24.37 (2.14)	24.44 (2.28)	987	24.52 (2.82)	23.86 (2.80)	76	25.16 (2.08)	25.63 (2.37)	270
*Muscle strength and endurance*											
Vertical jump (cm)	<0.001	1259	26.51 (5.41)	25.86 (5.70)	991	25.23 (5.54)	26.19 (4.88)	76	24.21 (4.96)	23.66 (5.34)	268
Accelerometer data											
% who meet the PA recommendation (60 min MVPA/day)	0.29	225	91.02	96.70	169	100	85.71	11	91.30	90.90	45

^a^The overweight group includes overweight and obese adolescents according to the IOTF criteria [30]

^b^ *P*-value for differences between overweight, normal-weight and underweight groups

The analysis was adjusted for the study design

**Table 3:** Baseline characteristics by fitness status (fit and low fitness)^a^

	*P* ^b^	All	Fit			Low fit		
		*n*	Control Mean (SD)	Intervention Mean (SD)	*n*	Control Mean (SD)	Intervention Mean (SD)	*n*
Age	0.86	1313	12.84 (0.71)	12.86 (0.72)	284	12.90 (0.84)	12.79 (0.78)	1029
Body mass index (kg/m^2^)	<0.001	1313	17.96 (1.80)	17.74 (1.84)	284	20.24 (2.92)	20.30 (3.41)	1029
Body mass index z-score	<0.001	1313	−0.22 (0.84)	−0.32 (1.04)	284	0.47 (0.97)	0.46 (1.07)	1029
Low socio economic status (%)	0.29	1260	34.39	35.90	274	31.84	31.29	986
Female proportion (%)	<0.001	1313	16.56	13.22	284	72.30	78.62	1029
Fitness (EUROFIT)								
*Speed*-*agility*								
Speed shuttle run (s)	<0.001	1310	23.20 (1.89)	22.72 (1.72)	284	24.96 (2.10)	25.08 (2.25)	1026
*Muscle strength and endurance*								
Vertical jump (cm)	<0.001	1304	28.22 (5.67)	28.89 (5.89)	284	25.29 (5.06)	24.67 (5.30)	1020
Accelerometer data								
% who meet the PA recommendation (60 min MVPA/day)	0.79	219	93.75	90.00	52	90.00	95.87	167

^a^The low fit were adolescents who did not reach the health zone according to the FITNESSGRAM standards [31]

^b^ *P*-value for differences between fit and low fit groups

The analysis was adjusted for the study design

### Intervention effects by BMI status

The intervention effect according to the BMI status is presented in Table 4. There were differential intervention effects for speed shuttle run (*P*_*i*_ = 0.06) between BMI groups. The intervention effect for adolescents with normal weight was β = −0.35 s [−1.63; 0.93]; β = −1.66 s [−6.31; 2.97] for underweight adolescents and β = −1.85 s [−2.59; −0.43] for overweight adolescents, i.e. the highest intervention effect was observed in the overweight group. Furthermore, this difference in intervention effect was significant only for the group of overweight adolescents (*P* = 0.04), which was independent of cardiopulmonary fitness, socio economic status and gender (*P*_*i*_ > 0.1 for all interaction terms) [34].

**Table 4:** Effect of the intervention according to BMI status

	All	Control	Intervention	Adjusted		Unadjusted	
	*n*	Mean (DS)	Mean (DS)	β [95 % CI]	*P* ^b^	β [95 % CI]	*P* ^d^
Fitness (EUROFIT)							
*Speed*-*agility*							
Speed shuttle run (s)					0.06^c^		0.08^e^
Normal weight	723	2.65 (3.37)	1.96 (2.41)	−0.35 [−1.63; 0.93]	0.59	−0.58 [−1.45; 0.28]	0.19
Underweight	60	2.72 (3.79)	2.61 (1.93)	−1.66 [−6.31; 2.97]	0.48	−0.20 [−1.91; 1.52]	0.82
Overweight^a^	188	2.85 (3.71)	1.34 (2.40)	−1.85 [−3.62; −0.08]	0.04	−1.51 [−2.59; −0.43]	0.006
*Muscle strength and endurance*							
Vertical jump (cm)		987	0.07 (6.45)	1.98 (6.80)		0.59^c^	0.85^e^
Accelerometer data							
% who meet the PA recommendation (60 min MVPA/day)		130	−18.09	−5.87		0.46	0.57

^a^The overweight group includes the obese adolescents

^b^ *P*-value adjusted for gender, socio economic status, fitness and all interaction terms between covariates and allocation group

^c^ *P*-value of interactions terms of BMI status (normal weight, underweight and overweight) X allocation group (control/intervention) after adjusting for gender, socio economic status, fitness and including all interactions between covariates and allocation group

^d^ *P*-value of the unadjusted analysis

^e^ *P*-value of the interaction term between BMI status (normal weight, underweight and overweight) X allocation group (control/intervention) from unadjusted analysis

There was no evidence that the intervention effects on vertical jump (*P*_*i*_ = 0.59) or in the proportion of adolescents who reached the recommendation of 60 min of moderate to vigorous physical activity (*P*_*i*_ = 0.46) were different amongst BMI groups.

### Intervention effects by fitness status

There were differential intervention effects for vertical jump (*P*_*i*_ = 0.02) between fitness groups (Table 5). The intervention effect for fit adolescents was β = 1.28 [−1.77; 4.32] cm and β = 3.71 [1.15; 6.28] cm for low-fitness adolescents which was significant for the later (*P* = 0.005) independently of BMI Z-score, socio economic status and gender (*P*_*i*_ > 0.1 for all interaction terms) [34]. No consistent differences between fit and low-fitness group were found for the intervention effect for speed shuttle run (*P*_*i*_ = 0.60) and for the proportion of adolescents who reached the recommendation of 60 min of moderate to vigorous physical activity (*P*_*i*_ = 0.94).

**Table 5:** Effect of the intervention according to fitness status

Outcomes	All	Control	Intervention	Adjusted		Unadjusted	
	*n*	Mean (DS)	Mean (DS)	β [95 % CI]	*P* ^a^	β [95 % CI]	*P* ^c^
Fitness (EUROFIT)							
*Speed*-*agility*							
Speed shuttle run (s)	971	2.69 (3.44)	1.89 (2.40)		0.60^b^		0.39^d^
*Muscle strength and endurance*							
Vertical jump (cm)					0.02^b^		0.15^d^
Fit	219	2.23 (6.73)	3.69 (7.19)	1.28 [−1.77; 4.32]	0.41	-	-
Low fit	768	−0.54 (6.22)	1.58 (6.66)	3.71 [1.15; 6.28]	0.005	-	-
Accelerometer data							
% who meet the PA recommendation (60 min MVPA/day)	130	−18.09	−5.87		0.94		0.30

^a^ *P*-value adjusted for BMI z-score, gender, socio economics status and all interaction terms between covariates and allocation group

^b^ *P*-value of the interactions terms of fitness status (fit/low fitness) X allocation group (intervention/control) after adjusting for BMI z-score, gender, socio economic status and including all interactions between covariates and allocation group

^c^ *P*-value of the unadjusted analysis

^d^ *P*-value of interaction term between of fitness status (fit/low fitness) X allocation group (intervention/control) from unadjusted analysis.

### Sensitivity analysis

The unadjusted model showed that the intervention effect on vertical jump was not significant different between fit and low-fitness (*P*_*i*_ of the allocation group x fitness groups = 0.15) in contrast to what was observed for the adjusted analysis (*P*_i_ = 0.02). The intervention effect on speed shuttle run according to BMI groups was similar for the unadjusted and adjusted analyses. After imputing missing values (*n* = 282/1440 for vertical jump and *n* = 286/1440 for speed shuttle run), the intervention effect on vertical jump decreased by 3.8 % (from β = 3.71, *P* = 0.005 to β = 3.57, *P* = 0.06) in low-fitness adolescents. For the BMI groups, the intervention effect on speed shuttle run became non-significant in overweight adolescents, changing from *P* = 0.04 β = −1.85 to *P* = 0.09 β = −1.58.

## Discussion

Our findings suggest that low-fit and overweight adolescents respond differently to ACTIVITAL program for two fitness outcomes compared to the fit and normal/underweight groups, respectively. Adolescents with poor physical fitness showed a higher improvement of muscular strength (vertical jump) compared to fit adolescents, after the intervention program. Whilst, overweight adolescents had a significantly lower increase in the time needed for speed shuttle run test compared to normal-weight and underweight adolescents i.e. although there was an overall decline in speed fitness with the time, this decline was smaller in the overweight adolescents compared to the normal-weight and underweight adolescents. These potential health benefits among adolescents at health risk (low-fit, overweight) are independent of the differences between weight and fitness groups in terms of age, socio-economic status, BMI and proportion of females. The latter is supported by the fact that our analyses were adjusted for all interaction terms between covariates and intervention allocation.

The findings of our analysis show that the intervention could provide positive effects on health [3, 35] among low-fit adolescents as they showed larger improvements on muscular strength compared to fit ones. Muscular strength and cardiorespiratory fitness are independently associated with NCD risks factors and are important determinants of general health during adolescence [3].

It has been reported that overweight adolescents have a lower performance on speed shuttle run than their normal peers, diminishing their self-efficacy, enjoyment for sport participation and physical exercise [36, 37]. Speed/agility is an independent predictor of bone mineral density in a young population and therefore, a persistent pattern of being slower and less agile through adolescence could compromise bone health at a later stage [3]. We consider that the intervention effect reported in the present manuscript is encouraging for overweight/obese adolescents in terms of speed shuttle run with a possible positive effect on bone health. However, we acknowledge that the absence of an effect in the underweight group could be a result of its smaller sample size [38] compared to the size of overweight and normal weight groups.

To our knowledge, only one Swiss study has examined simultaneously the intervention effect by weight category and fitness levels group among preschoolers. This study showed that low-fit and overweight preschoolers had a higher decrease in waist circumference and index of skin fold compared to fit and normal weight peers. In addition, the motor fitness improved more in overweight preschoolers compared to normal-weight ones [13]. Some studies have examined the variation of intervention effect only for fitness status or only for BMI status. As far as we know, only one study has analyzed the modification of intervention effect by fitness levels. This study reported the intervention effect on BMI, body fat and waist circumference by fitness levels in a population aged from 6 to 12 years. The latter study did not find that the intervention was more effective among low-fit subjects compared to fit ones [14]. On other hand, the modification of intervention effect only by weight category has been evaluated in some studies, but only in a younger population than that of the present study [9–12]. In most of those studies, the intervention effect on fitness (cardiorespiratory and muscle strength) was higher in the overweight group compared to normal weight group [9–12].

Our intervention was developed using a participatory approach, i.e. participants were involved in the health research process in order to improve the quality of the research design [17]. We speculate that this approach has created an environment in which the participants feel more comfortable and willing to be part of the intervention activities. In addition, our intervention used simple and positive messages that responded to a latent need for fun activities that address healthy lifestyles, in particular in the group of overweight and low-fit adolescents [39, 40]. This might have induced a higher response from these groups of adolescents that commonly face discriminatory attitudes and behavior at school [41]. Interventions that use high intensity physical activity or fitness sessions might have led to poor adherence in overweight/obese or low-fit adolescents due to their limited ability to carry out many of these activities [37]. Another plausible explanation, for the observed intervention effect among adolescents at health risk, is the presence of a “flooring effect”. Given their poor performance on the fitness components, the group of overweight or low-fit adolescents might have had more room and could require less effort to improve the motor fitness or speed/agility fitness components compared to normal weight [42] or fit adolescents [43, 44].

The results of the present manuscript suggest that future similar programs should consider the BMI or fitness status of adolescents if they expect to improve the speed—agility or muscular strength component among adolescents. Whilst, similar approaches could have an effect of physical activity independently of the BMI or fitness status of the adolescents. Besides, while the ACTIVITAL program is potential benefit for adolescents at high risk in terms of physical fitness only, more research are needed in order to identify approaches able to improve also the physical activity and screen-time behavior among overweight or low-fit adolescents. Additionally, future studies should invest time on sub-groups analysis in order to identify possible benefits among groups at health. This will provide sufficient evidence to confirm our findings.

Our results are limited to the populations with similar characteristics to Ecuadorian adolescents i.e. mixed *mestizo* ethnicity, living in urban areas at high altitude, with an obesity/overweigh prevalence around 22 %, a high proportion (>90 %) of adolescents that met the physical activity recommendations [24] and a high proportion (>60 %) of adolescents with low-fitness. However, the systematic process to develop the program could be generalized to adolescent populations with different prevalence of obesity/overweight/low-fit or with low proportion of adolescents that met the physical activity recommendations, since the approach used takes into account the needs and opinions of the participants to design the intervention. And therefore, the resulted intervention program might have an increased acceptability among the target groups.

### Strengths and limitations

Our study holds important strengths. First, our program uses a strong experimental design, with a sample size that is much larger than the average sample size (*n* = 300 participants) of similar school based studies in LMICs. A second strength is the duration of our program, which is longer than most trials focusing on a similar topic. Third, we used objective and clinically relevant cut-points (FITNESSGRAM standards) to classify adolescents into the fit and low fitness groups. Some limitations must be addressed. First, we used the 20 m shuttle run as an indirect measurement of VO_2_ max. We acknowledge that the estimation of VO_2_ max from results of 20 m shuttle run can vary according to the equation used. Nevertheless, in this study we used an equation [32] that has showed the highest agreement between the actual VO_2_ max and the estimate VO_2_ max from the 20 m shuttle run scores considering the age, gender and BMI of the subjects. Second, the dose received could not be assessed by the weight status or the fitness status of the adolescents, as the tools used to estimate the uptake only collected general information for the entire sample [23]. Third, physical activity was assessed only in a sub-sample. Finally, although our results are encouraging for school interventions in LMICs, our findings are modest and should be combined with other strategies to improve its effectiveness.

## Conclusions

Our results suggest that comprehensive school-based interventions to improve diet and physical activity could be beneficial in low-fit and overweight/obese adolescents who are already at health risk. The overweight/obese and low-fit adolescents responded differently to the intervention program compared to their normal/underweight and fit peers for the speed component (speed-shuttle run) and muscle strength component (vertical jump test) of the EUROFIT test. Future school interventions should consider the effect of their interventions strategies on the high-risk groups.

## Findings

This work was supported by grant from VLIR-UOS and Nutrition Third World and conducted within the cooperation between the University of Cuenca (Ecuador) and Ghent University (Belgium).

## Additional file

Additional file 1: Table S1. Mean and range of fitness scores at baseline, by BMI^a^ and fitness^b^ status. (DOCX 13 kb)

## Abbreviations

BMI: body mass index
LMICs: low-and middle-income countries
NCDs: noncommunicable diseases
