# Supplementary material for: Two years of school-based intervention program could improve the physical fitness among Ecuadorian adolescents at health risk: subgroups analysis from a cluster-randomized trial
Source: BMC Pediatr. 2016 Apr 22;16:51. doi: 10.1186/s12887-016-0588-8 (PMC4840972; doi:10.1186/s12887-016-0588-8)
Supplement: Additional file 1: Table S1. — Mean and range of fitness scores at baseline, by BMIa and fitnessb status. (DOCX 13 kb) [file 12887_2016_588_MOESM1_ESM.docx]

**Additional file 1: Table S1. Mean and range of fitness scores at baseline, by BMI ^a^ and fitness ^b^ status**

|  |  | ***Speed-agility*** | | ***Muscle strength and endurance*** | |
| --- | --- | --- | --- | --- | --- |
|  |  | **Speed shuttle run (s)** | | **Vertical jump (cm)** | |
|  |  | **n** | **Mean(range)** | **n** | **Mean(range)** |
| **Normal weight** | ***Control*** | 507 | 24.37 (19.00 - 33.60) | 508 | 26.51 (11.00 - 42.00) |
|  | ***Intervention*** | 480 | 24.44 (19.10 – 33.50) | 483 | 25.86 (7.00 – 48.00) |
| **Underweight** | ***Control*** | 32 | 24.52 (20.70 – 34.60) | 32 | 25.23 (14.50 – 37.00) |
|  | ***Intervention*** | 44 | 23.86 (19.80 – 29.00) | 44 | 26.19 (16.00 – 40.00) |
| **Overweight** | ***Control*** | 133 | 25.16 (21.40 – 33.20) | 133 | 24.21 (13.00 – 40.00) |
|  | ***Intervention*** | 137 | 25.63 (22.10 – 38.00) | 135 | 23.66 (6.00 – 38.00) |
|  |  |  |  |  |  |
| **Fit** | ***Control*** | 163 | 23.20 (19.00 – 34.60) | 163 | 28.22 (15.00 – 42.00) |
|  | ***Intervention*** | 121 | 22.72 (19.10 – 29.00) | 121 | 28.89 (13.50 – 48.00) |
| **Low fit** | ***Control*** | 489 | 24.96 (19.10 – 33.60) | 489 | 25.29 (11.00 – 42.00) |
|  | ***Intervention*** | 537 | 25.08 (20.10 – 38.00) | 536 | 24.67 (6.00 – 43.00) |

^a^ The overweight group includes overweight and obese adolescents according to the IOTF criteria

^b^ The low fit were adolescents who did not reach the health zone according to the FITNESSGRAM standards
